# Neurobiological Degeneracy and Affordance Perception Support Functional Intra-Individual Variability of Inter-Limb Coordination during Ice Climbing

**DOI:** 10.1371/journal.pone.0089865

**Published:** 2014-02-24

**Authors:** Ludovic Seifert, Léo Wattebled, Romain Herault, Germain Poizat, David Adé, Nathalie Gal-Petitfaux, Keith Davids

**Affiliations:** 1 CETAPS Laboratory - EA 3832, Faculty of Sports Sciences, University of Rouen, Rouen, France; 2 LITIS Laboratory - EA 4108, National Institute of Applied Sciences (INSA), Rouen, France; 3 Faculty of Psychology and Educational Sciences, Department of Adult Education, University of Geneva, Geneva, Switzerland; 4 ACTÉ Laboratory - EA 4281, Faculty of Sports Sciences, University of Clermont-Ferrand 2, Clermont-Ferrand, France; 5 Centre for Sports Engineering Research, Sheffield Hallam University, Sheffield, South Yorkshire, United Kingdom; West Virginia University School of Medicine, United States of America

## Abstract

This study investigated the functional intra-individual movement variability of ice climbers differing in skill level to understand how icefall properties were used by participants as affordances to adapt inter-limb coordination patterns during performance. Seven expert climbers and seven beginners were observed as they climbed a 30 m icefall. Movement and positioning of the left and right hand ice tools, crampons and the climber’s pelvis over the first 20 m of the climb were recorded and digitized using video footage from a camera (25 Hz) located perpendicular to the plane of the icefall. Inter-limb coordination, frequency and types of action and vertical axis pelvis displacement exhibited by each climber were analysed for the first five minutes of ascent. Participant perception of climbing affordances was assessed through: (i) calculating the ratio between exploratory movements and performed actions, and (ii), identifying, by self-confrontation interviews, the perceptual variables of environmental properties, which were significant to climbers for their actions. Data revealed that experts used a wider range of upper and lower limb coordination patterns, resulting in the emergence of different types of action and fewer exploratory movements, suggesting that effective holes in the icefall provided affordances to regulate performance. In contrast, beginners displayed lower levels of functional intra-individual variability of motor organization, due to repetitive swinging of ice tools and kicking of crampons to achieve and maintain a deep anchorage, suggesting lack of perceptual attunement and calibration to environmental properties to support climbing performance.

## Introduction

At elite performance levels, such as in high-level sports, expertise is expressed by individuals achieving consistent performance outcomes, often in highly dynamic performance environments. According to Ericsson and Lehman [1], an expert is defined as an individual with at least ten years or 10000 hours of deliberate practice. They argued that expertise results from repeated, motivated engagement in an activity requiring effort and concentration, both of which have little to do with talent [1]. In cognitivist approaches, a high level of expertise results in the capacity to reproduce a movement like an automatism, with movement variability considered as a dysfunctional aspect of motor control, representing the amount of noise to be reduced for achievement of successful performance [2]–[5].

Alternatively, in an ecological dynamics approach [6], [7], it has been suggested that the nature of performer-environment relationship and the coupling of perception and action is not the same for beginners and experts. This is because the expert is more capable of exploiting information about environmental and task-related constraints to functionally (re)organize the multiple degrees of freedom of the body to achieve consistent performance outcomes. Thus, the greater adaptability of the experts to a variety of interacting constraints, categorised as organismic, environmental and task-related [8], has emphasized the functional role of movement variability [3], [9], [10]. From this perspective, movement variability allows the performer to explore different perceptual-motor solutions, facilitating the discovery of functional patterns of coordination and is supported by inherent neurobiological system degeneracy [10]–[12]. According to Edelman and Gally [12], “*degeneracy is the ability of elements that are structurally different to perform the same function or yield the same output*” (pp. 13763). It signifies that elements of the system exhibit heteromorphy and also iso-functionality [13]. With regards to the process of skill acquisition, degeneracy signifies that, with practice, a learner can structurally vary his/her perceptual-motor system organization without compromising function, supporting the adaptive and functional role of movement pattern variability in satisfying task constraints. In complex performance environments, therefore, it could be hypothesized that expert athletes are able to pick up several sources of information (visual, haptic, acoustic, etc), to perform various types of actions and/or to use co-existing modes of coordination (i.e., exploiting perceptual-motor system multi-stability and meta-stability; [14], [15]) to achieve the same functional performance outcomes. Multi-stability refers to the capacity to transit between multiple states of organisation under specific performance constraints (for an example of co-existing in-phase and anti-phase coordination patterns of finger oscillation when moving at a given frequency, see [16]). Meta-stability corresponds to the capacity to exploit co-existing coordination tendencies in a transition or unstable region of a complex movement system [15]. This latter idea has been supported in observations of athletes training without specific instructions on which coordination patterns to use in achieving their task goals. For example, Hristovski et al. [17], [18] investigated how boxers’ striking patterns were adapted when they punched a heavy bag at various distances. They showed that, at great distances from the boxing bag, a ‘jab’ movement pattern tended to emerge, whereas at close distances, ‘uppercuts’ or ‘hooks’ patterns were more typically observed. Finally, a critical intermediate distance from the boxing bag seemed to represent a meta-stable performance region where a varied, rich and creative range of movement patterns emerged, such as ‘uppercuts’, ‘hooks’ and ‘jabs’ [17], [18]. Thus, in a meta-stable performance region one or several movement patterns are weakly stable (when there are multiple attractors) or weakly unstable (when there are only attractor remnants), and switching between two or more movement patterns occurs under interacting performance constraints [15], [19]. Kelso [15] noted that, in a meta-stable region, there is attractiveness, but strictly speaking no attractors. This evidence suggests that multi- and meta-stability of perception and action can reflect degeneracy in neurobiological complex systems.

These data are aligned with theoretical ideas from ecological dynamics. According to the insights of Gibson [20], who defined affordances as opportunities for action offered by the environment, the previous data suggests that the boxing striking patterns transitioned according to the perception of a ‘strike-ability’ affordance (i.e. the perception of the distance to a target which afforded specific striking actions to emerge) [17], [18]. Although different conceptions of affordances exist in the literature [21]–[26], ecological psychologists assume a direct perception (i.e., perception which is not mediated by prior knowledge and assumptions about the world) expressed and, in turn intrinsically linked to actions. In the sport of rock climbing, Boschker et al. [27] previously analysed the perceived structural features of climbing wall and climbing opportunities (also defined ‘climbing’ affordances by the authors). The results showed that unlike beginners, expert rock climbers neglected to perceive the structural features of a climbing wall, but they recalled more information than novices and focused on its functional properties, displaying greater exploitation of climbing affordances. Rock climbing affordances could refer to environmental properties, which invite hold reach-ability, grasp-ability and climb-ability. Previous research has shown that experts generally display better perceptual attunement to and calibration of informational variables, in specific performance environments, notably because they do not tend to rely on similar informational variables than novices in a specific activity. In particular, it has been reported that experts tend to rely on a range of perceptual variables of different modalities that specify a relevant property of a performance environment that they seek to perceive [21], [28]. In this respect, the term ‘relevant’ signifies functionality as this property enables an individual performer to achieve a specific task goal with efficacy.

In the current study, we examined the relationship between movement pattern variability and the perception of affordances in climbers of different skill levels. We sought to establish whether expert climbers could pick up climbing affordances by exploiting available environmental properties with higher levels of adaptive movement pattern variability than beginners. If this relationship were observed it might reveal the functional role of degeneracy, a key property of complex, neurobiological systems.

Ice climbing is a rich task to examine how differing participant skill levels can provide insights on the relationship between affordances detection and neurobiological system degeneracy. Ice climbing involves quadruped locomotion as performers ascend a vertical frozen waterfall surface with ice tools in each hand and crampons on each foot. Only the extremities of these tools are anchored in the frozen waterfall surface [29]. Moreover, although ice climbers attempt to determine their own climbing paths, skilled coordination of upper and lower limbs emerges from the interaction of each performer with the specific properties of the icefall during ascent. This is because specific modes of coordination and the condition of the ice are not completely predictable from ground level prior to climbing. Indeed, key properties of the icefall (shape, steepness, temperature, thickness and ice density) vary stochastically through the ascent, dependent on specific weather patterns, the ambient temperature throughout the climb (when a part of the icefall switches from the dark side to sunny side) and the altitude of the location. These environmental properties are not completely under the control of the climber, which probes the skills of each individual. The climbing task requires successful performers to actualise the relationships between their intentions, perceptions and actions through their ascent. Successful relations between these processes could be exhibited by exploiting the neurobiological property of degeneracy, for instance by using numerous different types of action (e.g., swinging, kicking and hooking) and varied patterns of inter-limb coordination (e.g., horizontal-, diagonally- and vertical-located angular positions of arms and legs) during performance. Moreover, evidence for perception of affordances when climbing could be provided by observations of the climbers swinging their ice tools to create their own holes to support their weight or perceiving and hooking existing holes (due to the actions of previous climbers or by exploiting the presence of natural holes in the ice fall surface).

An important aim of this study was to analyse the inter-limb coordination patterns of ice climbers, differing in skill level, in order to investigate how existing properties of an icefall can provide climbing affordances for participants by exploiting their inherent neurobiological system degeneracy during performance. We assumed that the reverse causality is also true, predicated on the circular coupling between perception and action. Therefore, another aim was to investigate how the active exploration of icefall properties can lead performers to rely on relevant and functional properties of the performance environment to achieve their task goals. First, it was hypothesized that expert climbers would be able to exploit a larger range of coordination patterns and types of movement (e.g., swinging, kicking and hooking) than beginners, reflecting multi-stable behaviour and supporting neurobiological degeneracy. Second, we expected that experts would be able to detect various sources of regulating information (e.g., size, depth and shape of the holes in icefalls), specifying a range of functional actions, whereas beginners would tend to perceive global, structural icefall characteristics (e.g., existing holes in icefall), which may not specify actions. In summary, we hypothesized that in exploiting degeneracy, expert climbers were also opening up more opportunities to perceive affordances and vice versa, suggesting that affordance perception and neurobiological degeneracy may evolve together during skill acquisition.

## Methods

### Participants

Fourteen, male ice climbers, differentiated into two groups, volunteered to participate in this study. One group included seven expert climbers with mean age: 32.1±6.1 yr; mean height: 176.4±6.2 cm; mean weight: 68.4±6.7 kg; skill level in rock climbing: grade 7a+ to 7c on the French rating scale (ranging from 1 to 9); mean number of years practising rock climbing: 17.1±5.6; skill level in icefall climbing: grade 6 to 7 on the French rating scale (ranging from 1 to 7) [29]; mean number of years of practice ice climbing: 10.4±4.7; mean number of days of ice climbing per year: 20.6±9.3. They were considered at the skill stage of motor learning [30] since they were: (i) mountain guides, certified by the International Federation of Mountain Guides Association (IFMGA) or/and (ii) instructors at the French National School of Skiing and Alpinism (ENSA). The other group included seven beginners (mean age: 29.4±6.8 yr; mean height: 176.1±6.1 cm; mean weight: 71.1±9.8 kg) who were students in a faculty of sport sciences at a local university, with 20 hours of practice in artificial wall climbing and were inexperienced at ice climbing. They can be considered at the coordination stage of learning [30].

### Protocol

To impose a similar task constraint on both groups [8], a sub-maximal level of effort was required that corresponded to a 30 m icefall climb at grade 5+ for expert climbers (which is a regular grade for them). The beginners climbed a 30 m icefall at grade 4 (a common grade assigned to that skill level). Grade 5+/6 signifies vertical climbing for most of the icefall, while grade 4 involves alternation of steep sections around 80 to 85° with ramps around 60–70°. For this research protocol, the icefall selected for the beginners was in three sections: 20 m at 85°, ramp of 5 m at 70°, then 5 m at 80°. Although a similar task constraint was imposed on the participants, these differences of grade between the two groups represented different environmental constraints (i.e. in terms of steepness). Consequently, to enable a valid comparison between skilled climbers and beginners, only performance of both groups on the first 20 m section of the ice fall, corresponding to 85° of steepness, was selected for analysis of their motor behaviours. Performance data were collected in two sessions during which the air temperature was respectively −8°C and −12°C. All climbers were equipped with the same crampons and ice tools and were instructed to climb at their normal pace.

### Ethics Statement

The protocol was approved by the Rouen University ethics committee and followed the Declaration of Helsinki. Procedures were explained to the participants, who then gave their written informed consent to participate.

### Data Collection

For movement analysis, a frontal full HD camera (Canon HF-G25, 1920×1080 pixel, used with a frame rate frequency of 25 Hz), positioned 15 m behind the climber and perpendicular to the icefall, digitally recorded the first 20 m of the climb. A calibration frame delimited the recorded space of climbing performance and was composed of one vertical rope with marks every 2 m and two horizontal ropes (at 5 m and at 20 m) with marks every 1 m (total of 20 marks for calibration). Five key points (the pelvis, the head of the left and right hand ice tools and the extremity of left and right foot crampons) were digitised using Simi Motion Systems® (2004). When four repetitive digitisations of the same individual were performed, the average error of manual digitizing for X and Y coordinates of each ice tool and crampon was assessed by calculating the root-mean-square (RMS in cm) and the coefficient of variation (CV in %). The calculated error was: in X RMS = 3.5 cm and CV = 2.4%, in Y RMS = 3.8 cm and CV = 2.7%.

For identifying perceptual variables that were meaningful for the climbers, we were inspired by the protocols of Boschker et al. [27], who analysed affordances in rock climbing and the perceived structural features of a climbing wall using a ‘think-aloud’ method to gather verbalisation data. In that study, participants were invited to verbally report everything they thought about, especially what was perceived when looking at the climbing wall [27]. When participants showed difficulties in verbal recall, they were prompted to explain their actions and what they had perceived during route finding [27]. In our study, individual self-confrontation interviews were conducted with each participant no more than one hour after the ascent in order to collect verbalized data about each climber’s lived experiences during the climb. This interview method was developed by Von Cranach and Harré [31] and consists of confronting the participant with traces of his or her activity (notably the video recordings). Numerous recent empirical studies in the field of expertise in sports have demonstrated the fruitfulness of this methodology for studying the activity-situation coupling in athletes (e.g., [32]–[36]). Each climb was videotaped to provide a basis for the collection of the verbalized data. During each interview, the participant and researcher viewed the video recording of the climb (the interviews lasted for about one hour each). The participant was invited to describe and comment on his own lived experience step by step, which in our study concerned the perceptions (e.g., informational variables such as visual, kinaesthetic, haptic, acoustic variables), intentions and actions. Before each interview, the researcher/interviewer reminded the participant of the nature of the interview and the expectation that the participant needed to “re-live” and describe his own experience during his ascent, without an *a posteriori* analysis, rationalization or justification. This method is designed to reach the level of activity that is meaningful for the actor: the goal is to encourage the participants to verbally report what they did, felt, thought, and perceived during the climb, as naturally as possible. The self-confrontation interviews aimed to explore the coupling between perception, action and intentions of each participant based on a retrospective viewing of performance. The researcher used specific prompts to encourage the participant to re-experience the dynamic performance situation, that is, to assume an attitude and mental state favourable to expressing his or her experience. In order to eliminate pre-formed experiences, athletes were involved in an attitude of evocation. Athletes were asked not to repeat a theoretical description of their performance but to consider what they had specifically experienced during the ascent. Behaviour indicators such as hesitations in the stream of language, unstructured sentences or an introspective stare were used as indicators of evocation [37]. In the present study the interviews were conducted by three co-authors of this paper who had extensive experience of qualitative research and, specifically, of using self-confrontation interviewing techniques. The self-confrontation interviews are well-suited for our research purposes because: (i) researchers have access to the experiential dimension of activity, including climbers’ actions, intentions, and perceptions, (ii) activity can be studied by reconstructing the natural and sport-specific conditions of the climb, and (iii), activity can be accessed without interfering with performance (compared with the ‘think-aloud’ method which might have interfered with emergent perception-action coupling tendencies during performance).

### Data Analysis

#### 1. Performance outcomes and fluency of climbing movement

Since climbing was self-paced, the time of ascent was not considered as a performance outcome. Instead the first 20 m section of the icefall was used to gain a universal measure of performance outcome: the distance travelled by the pelvis of each participant in the vertical axis during a 5-minute period for both samples (since the frame rate of the camera was 25 Hz, *n* = 7500). Previous studies have assessed the fluency of climbing movements by conducting a harmonic analysis of the acceleration of the body’s centre of mass [38], by quantifying the duration of a static position as any point throughout the climb where the hips were not in motion [39] or by analysing the geometric entropy indices from the displacement of the body’s centre of mass [40]–[42]. Based on this previous work, we assessed the fluency of climbing movement by recording the average number of plateaux and average plateau duration from the vertical distance-time curve of the pelvis of each climber. A plateau was detected by an algorithm, which provided a moving window on the whole signal. Several iterations of the algorithm enabled us to empirically select the best threshold to discriminate the experts and beginners. In particular, when the difference between the two points delimiting the window was higher than a fixed threshold (in our case, vertical axis pelvis displacement lower than 0.15 m) a break point was detected in the window. The difference was a Euclidean distance (*d*) following this equation:
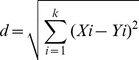



where X and Y are the two points delimiting the window. Moreover, another threshold (in our case, a plateau duration greater than 30 s) was selected in order to avoid doublets in the detection. If two break points were lower than this threshold value, the algorithm ignored the new break point detected. Finally, a plateau was considered as a vertical axis pelvis displacement less than the value of 0.15 m for durations longer than 30 s.

#### 2. Inter-limb coordination

**Figure 1:**
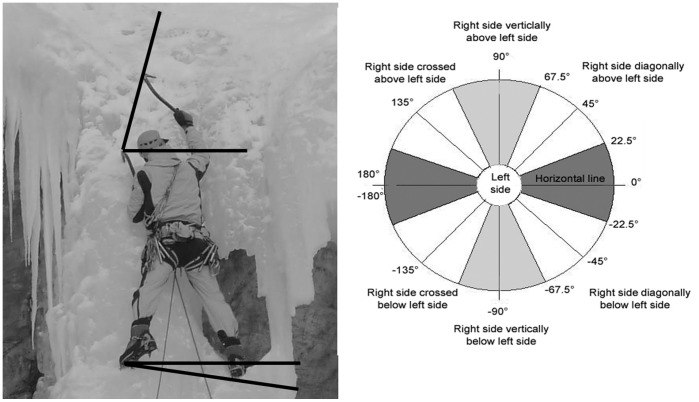
Angle between horizontal, left limb and right limb (left panel). Modes of limbs coordination as regards the angle value between horizontal, left limb and right limb (right panel).

Upper limb coordination patterns were assessed by using the angle between the horizontal line and the displacement of the heads of the left and right hand ice tools. Lower limb coordination patterns corresponded to the angle between the horizontal line and the displacement of the left and right crampons (Fig. 1). These two signals were smoothed by a Butterworth low-pass filter (cut-off frequency 6.25 Hz) by Matlab 7.7® (1984–2008, The MathWorks, Inc.). When the angle was 0±22.5°, the two limbs were horizontal, meaning that they were simultaneously flexed or simultaneously extended, corresponding to an in-phase mode of coordination. When the angle was +or- 90±22.5°, one limb was vertically located above the other limb, meaning that one was flexed, while the other was extended, corresponding to an anti-phase mode of coordination. Between these values, it was considered that coordination was in an intermediate mode. The angle between the horizontal line and the left and right limbs was positive when the right limb was above the left limb and negative when the right limb was below the left limb (Fig. 1).

The phase angles of the upper and lower limbs were obtained by Hilbert transform (Matlab 7.7 ® 1984–2008, The MathWorks, Inc.), usually calculated for non-periodic signals [43]–[46]:

Phase = arctan s(t)/*H*(t).with s(t) as the real part and *H*(t) the imaginary part of the signal. After Rosenblum and Kurths [47], *H*(t) was obtained as follows:



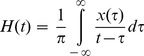
where *x*(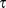) is a given time series. The relative phase 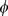
(t) between upper and lower limbs given in radians for two time series upper limbs *upper*(t) and lower limbs *lower*(t) is obtained by:


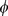
(t) = arctan [H_upper_(t) * s_lower_(t) – s_upper_(t) * H_lower_(t)]/[s_upper_(t) * s_lower_(t) – H_upper_(t) * H_lower_(t)]

An in-phase mode of coordination was assumed to occur for −22.5°<
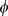
(t)<22.5° and meant that upper and lower limbs displayed similar angular locations. An anti-phase mode of coordination was taken to be between −180°<
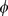
(t)<−157.5° and 157.5°<
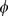
(t)<180° and meant that upper and lower limbs showed opposite angular locations (e.g., −90° for the upper limbs coordination and 90° for the upper limbs coordination). Between these values, inter limb coordination was considered to be in an intermediate mode and meant that upper and lower limbs showed a gap of >22.5° in their angular locations (e.g., 90° for the upper limbs coordination and 0° for the upper limbs coordination). Finally, mean and standard deviation data of 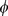
(t) were calculated throughout the 5-minute period for each individual.

#### 3. Types and frequency of ice tool and crampon actions

Different types of actions could be realised by individuals (i.e., swinging, kicking or hooking), depending on the icefall shape: when the ice is dense without any holes, climbers usually swing their ice tools and kick their crampons. Conversely, when the ice is hollow, climbers hook holes with their ice tools and crampons. Thus, the functional ability of each climber to vary the types of action used to engage with the dynamic properties of each specific icefall was assessed by counting the types of action from the video footage and two ratios were calculated: (i) the ratio between the actions of ice tool swinging and hole hooking, (ii) the ratio between the actions of crampon kicking and hole hooking.

Calculating the frequency of actions to anchor the ice tools could also reveal the ability of each climber to exploit and be attuned to icefall properties. Indeed, when the ice is soft or ventilated, climbers can anchor their ice tools and crampons in one shot. Conversely, when the ice is dense and thick, climbers need to repeat numerous trials of ice tool swinging and crampon kicking to attain a safe anchorage. Usually skilled climbers can detect the modification of the thickness of the icefall in order to minimize the frequency of actions they need to complete before definitive anchorage [48]. Based on these ideas, the frequency of actions used to anchor the tools was calculated from the video footage and two ratios were derived: (i) the ratio between repetitive ice tool swinging and definitive anchorage, and (ii) the ratio between repetitive crampon kicking and definitive anchorage. A similar performance indicator is already used in rock climbing to detect skill in climbing quickly up a vertical surface. In rock climbing, Pijpers et al. [49] distinguished between what they termed as *exploratory* and *performatory* movements according to whether a potential hold on the rock surface was touched, with or without it being used as support. The relation between exploratory and performatory movements could be analysed through the ratio of touched holds and grasped holds, for which climbing skills is usually defined by the ‘three-holds-rule’. Sibella et al. [42] have reported that skilled climbers can move quickly by using fewer than three holds, signifying that they had touched fewer than three surface holds before grasping the functional one.

#### 4. Climbers’ experience during ascent

The verbalisation data gathered during the self-confrontation interviews were processed according to the procedure defined in the course-of-action methodology [50]–[52]. This method of interview follows a comprehensive and idiosyncratic approach. The first step consisted of generating a summary table containing the data recorded during the climb (i.e., a brief description of the climber’s behaviours) and during the self-confrontation interview (i.e., verbatim transcriptions of the prompted verbalizations). The second step consisted of identifying the elementary units of meaning (EUMs), which are the smallest units of activity that are meaningful for an actor. This process was accomplished by analysing the audio-video recording and the verbalization transcripts. The third step consisted of reconstructing each climber’s personal experiences, leading to the identification for each EUM, of the perceptions, intentions and actions that were considered meaningful by each actor. It is worth pointing out that, although perceptions, actions and intentions are described in three separated columns in Tables 1 and 2, the self-confrontation interviews aimed to explore perceptions, actions and intentions as integrated and not as separated components of each participant’s activity. The fourth step consisted of identifying the typicality of participants’ experience. The typicality character refers to at least four aspects [53]: descriptive (i.e., the typical occurrence presents the highest number of attributes of the observed activity in the sample of actors and the studied situations), statistics (i.e., the typical occurrence is the most frequently observed in the studied sample), generative (i.e., the typical occurrence has a propensity to update when conditions having a resemblance to those observed are reproduced), significant (i.e., actors express a feeling of typicality when they are questioned about that during self-confrontation interview). From there, the comparison between and within climbers was based on these typical experiences and not from the data recorded from a pre-existing analysis grid. Several measures were taken to enhance the validity of these data [54]. First, the transcripts were returned to the participants so that they could ensure the authenticity of their commentary and make any necessary changes to correct the text. Second, the data were processed independently by two trained investigators. These researchers were experienced in conducting qualitative research, and familiar with the course of action methodology.

**Table 1:** Illustration of perceptions, actions and intentions of expert climbers.

Perceptions	Actions	Intentions
Good hole is **deep** and **vertically oriented**(e.g., expert 3: “*it must be a vertical hole”…* *“I try to find a deep hole, deeper is the hole,* *better it is*”).Good hole when the ice is **dense** and**homogeneous** around this hole. Perceptionfocussed on the **sound** of the tools againstthe icefall to detect information about theproperty of **ice thickness** (e.g., expert 3:“*it’s not only the hole, it’s also the ice* *quality… the manner of which the ice* *responses to us, the sound is very important,* *especially when the blade penetrates in the* *ice…a good sound is a short thud sound,* *not too loud…when there is no vibrations,* *the ice tool will anchor uniformly in the ice*”).	Good hole is a hole that could be **hooked** andnot swung into (e.g., expert 2: “*There is a hole,* *I know I can put the ice tool, just put the ice tool* *like this*”).Instead of automatically swinging their ice tools,experts put the **blade horizontally** into the holeand apply a **downward force** or they **smoothly** **whipped** the ice tool with thewrist (e.g., expert 3:“*I just apply a small wrist acceleration at the end* *of the hooking like a whipping*”).The **upper and lower limb actions** seemedstrongly linked because the holes used by the icetools were also exploited for the crampons (e.g.,expert 5: “*I tried to re-use the hole done by my* *ice tools for my crampons*”).	Focus on **safety**, since one of their goals was to **save the icefall structure** (e.g., expert 4: “*My goal is to swing with the weakest force to minimize damage of the icefall, because it remains a fragile structure*”).Focus on **efficiency** and **energy economy** by:**Hooking hole** (e.g., expert 1: “*The most economic strategy is to hook; so when I can, I just put my blade in a hole that is more economic than swinging. Ice tool swinging becomes rapidly tiring*”),**Balancing the body** (e.g., expert 3: “*I move my crampons step by step from right to left to centre my pelvis between my ice tool*”; expert 2: “*If I move too far from my ice tool, when I take of my other ice tool, my body will turn like a door, so I try to regulate my posture by moving my foot through small and numerous kicking*”),**Maintaining a constant climbing fluency and speed** (e.g., expert 5: “*I anchor my two ice tools, then I move my crampons step by step, and so on*”).

**Table 2:** Illustration of perceptions, actions and intentions of beginners.

Perceptions	Actions	Intentions
Good hole is **big** and**deep** (e.g., beginner 2:“*a good hole is a big* *and deep hole; if the* *hole is like 2 cm, I go* *on a side to see a deeper* *one exists*”).	A deep hole looks like a hole where the **blade** could be **fully** **anchored** (e.g., beginner 1: “*I look for a deep anchorage, with the blade* *half or fully anchored*”) and the **stick** of the ice tool is **close to the ice** **fall surface** (e.g., beginner 2: “*when I find a good hole like this, the glove* *touched the ice fall…when my hand is close to the ice fall, I’m confident* *in my anchorage*”).To anchor the blade, the beginners **swung** the blade in the holethen tried to pull it downward (e.g., beginner 3: “*I pull on the* *stick once the blade is anchored to test it*”).Beginners attempted to put their **crampons horizontally on the** **steps** (e.g., beginner 2: “*I put my foot like duck, horizontally on the step* *and I don’t especially take care of the two frontal peaks of the crampons*”).When they were not able to find steps, they engaged in **repetitive** **kicking with their crampons** until creating a big hole in the icefallto design a deep step (e.g., beginner 1: “*when there is no step or* *platform, I dig the icefall*”).	Focus on **safety** found only in:**Deep ice tool anchorage** (e.g., beginner 4: “*I’m scared when the ice tool is only put on the blade extremity. I looked for a deep anchorage with my ice tool because I feel it is better when the blade does not move*”),**Natural step** **with a large surface** on which to put their crampons,**Stabilised blade and ice tool that** **did not move** (e.g., beginner 3: “*When the ice tool is well anchored and touched the icefall, I keep going*”),**Short time** where they needed to use the **frontal peaks of the crampons** because they felt insecure and in an unsafe situation (e.g., beginner 3: “*When the icefall was vertical, only the frontal peaks of the crampons were anchored, it was like nothing, no support*”).

Finally, as previously undertaken in studies of rowing [55], swimming [56] and more globally, in performance analysis in sport [35], this description of typical experience was articulated with the quantitative data that we recorded on inter-limb coordination patterns, types and frequency of actions, and fluency of climbing movement to highlight their relationships.

### Statistical Analysis of the Data

The normality of the distribution (Ryan Joiner test) and the variance homogeneity (Bartlett test) of the data were checked before using parametric statistics. Two-way ANOVA (fixed factor: skill level; random factor: participant) compared the performance measure (pelvis displacement in the vertical axis), the upper limb angle, the lower limb angle and the upper-lower limb coordination pattern between the two skill groups. All tests were conducted with Minitab 15.1.0.0 ® software (Minitab Inc., Paris, France, 2006) with a conventional statistical significance level of p<0.05.

## Results

### Performance Outcomes and Fluency of Climbing Movement

Experts covered a greater distance on the vertical axis in 5 minutes than beginners (13.8±4.8 m *vs.* 7.2±3.9 m; F_1,6_ = 13.25, *p*<0.05). Analysis of the vertical displacement-time curve for the pelvis showed a greater number of plateaux for expert climbers than for beginners (10.2±0.5 *vs.* 8.0±1.6; F_1,6_ = 6.76, *p*<0.05), but of shorter time durations (28.5±2.1 s *vs.* 36.0±7.5 s; F_1,6_ = 7.48, *p*<0.05).

### Inter-limb Coordination

**Figure 2:**
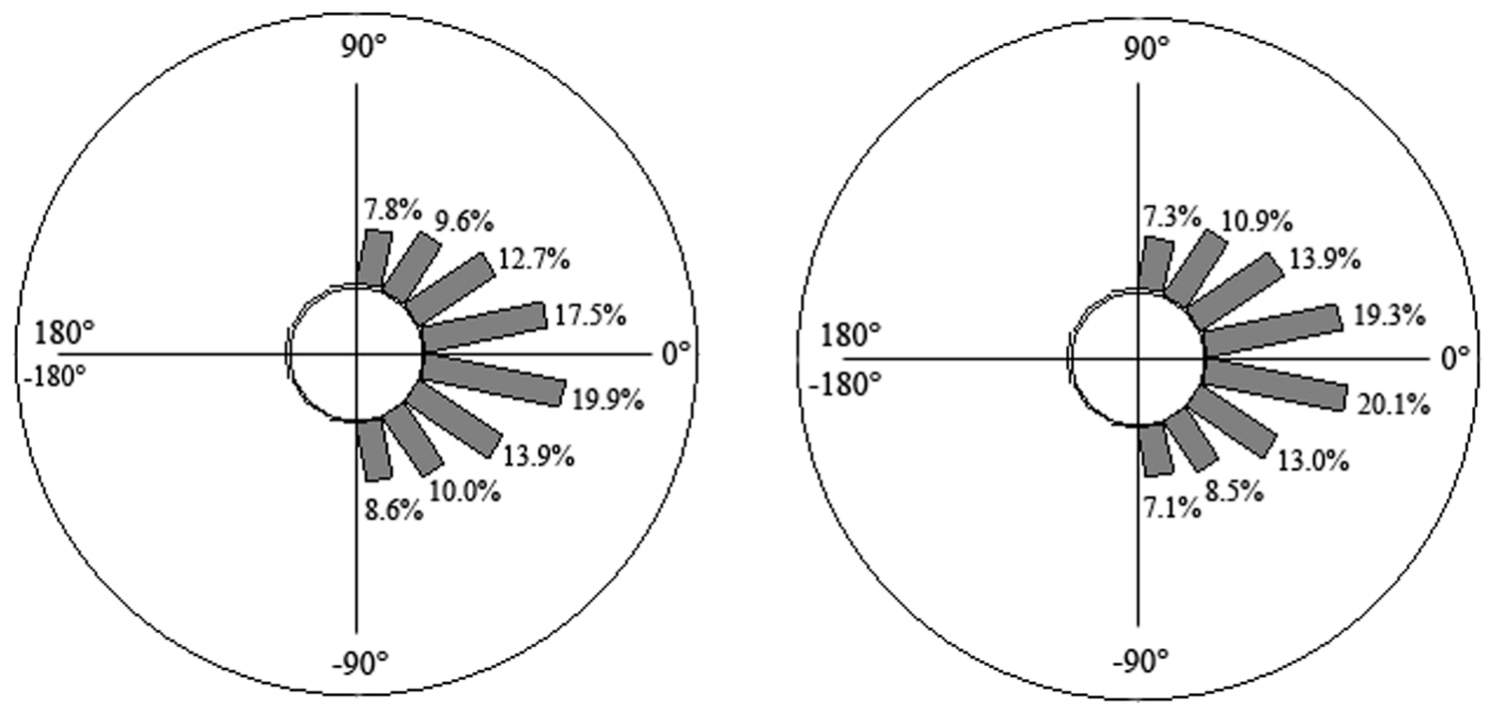
Upper-lower limb coordination: Time spent (in % of the climbing duration) with the upper and lower limbs in different coordination modes; left panel: expert climbers, right panel: beginners.

Both groups showed upper-lower limb coordination patterns, which were predominantly in an in-phase mode (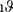
(t) = 1.7±3.8° for beginners and 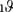
(t) =  −1.6±1.6° for expert climbers), but with a great variation in the coordination patterns observed during climbing (standard deviation of 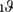
(t) = 42.5° for beginners and 44.8° for expert climbers). Notably, 39.4±13.3% of the time spent climbing was in an in-phase mode for beginners and 37.4±5.1% for expert climbers (Fig. 2). The remaining upper-lower limb coordination interactions varied between −90°<
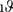
(t)<90°.

**Figure 3:**
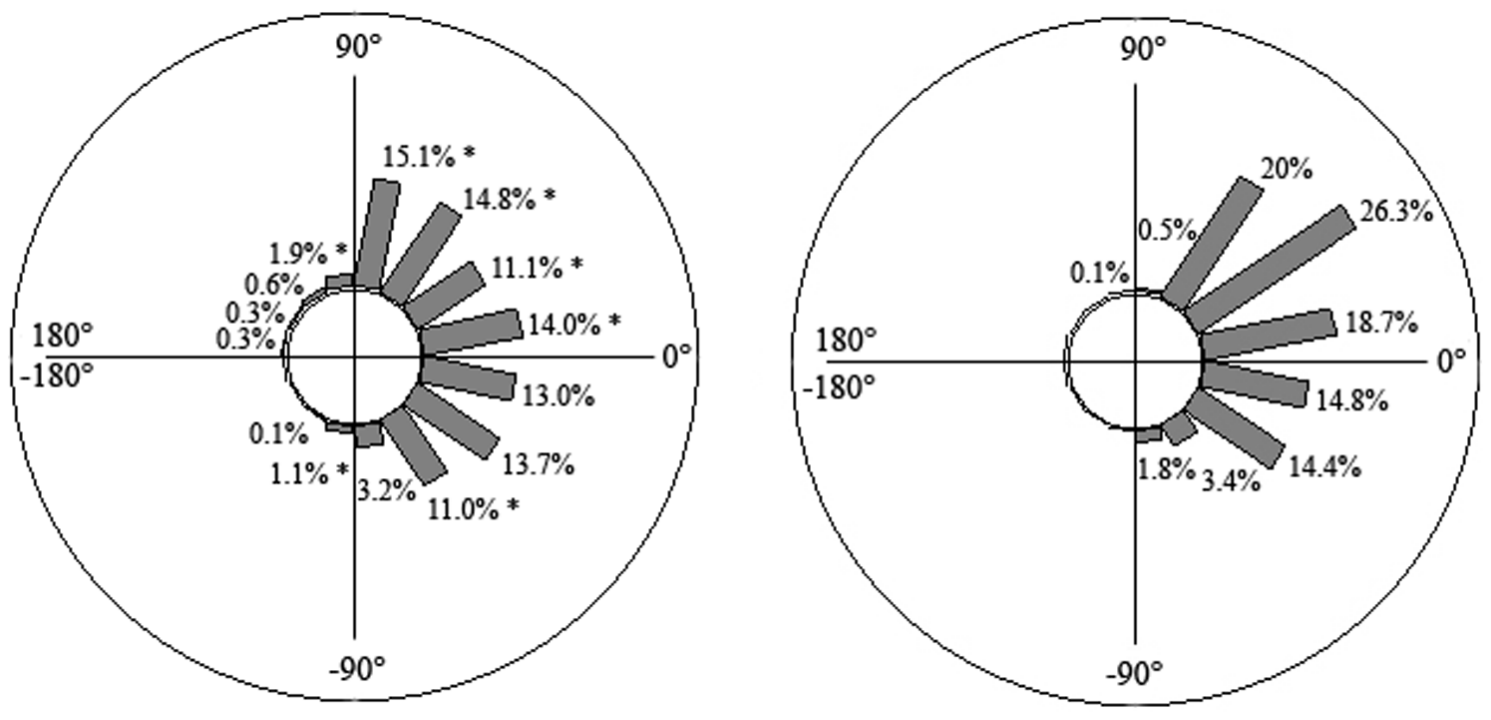
Upper limb coordination: Time spent (in % of the climbing duration) with the ice tools in different angular positions; left panel: expert climbers, right panel: beginners; *: significant differences with beginners at *p*<0.05.

**Figure 4:**
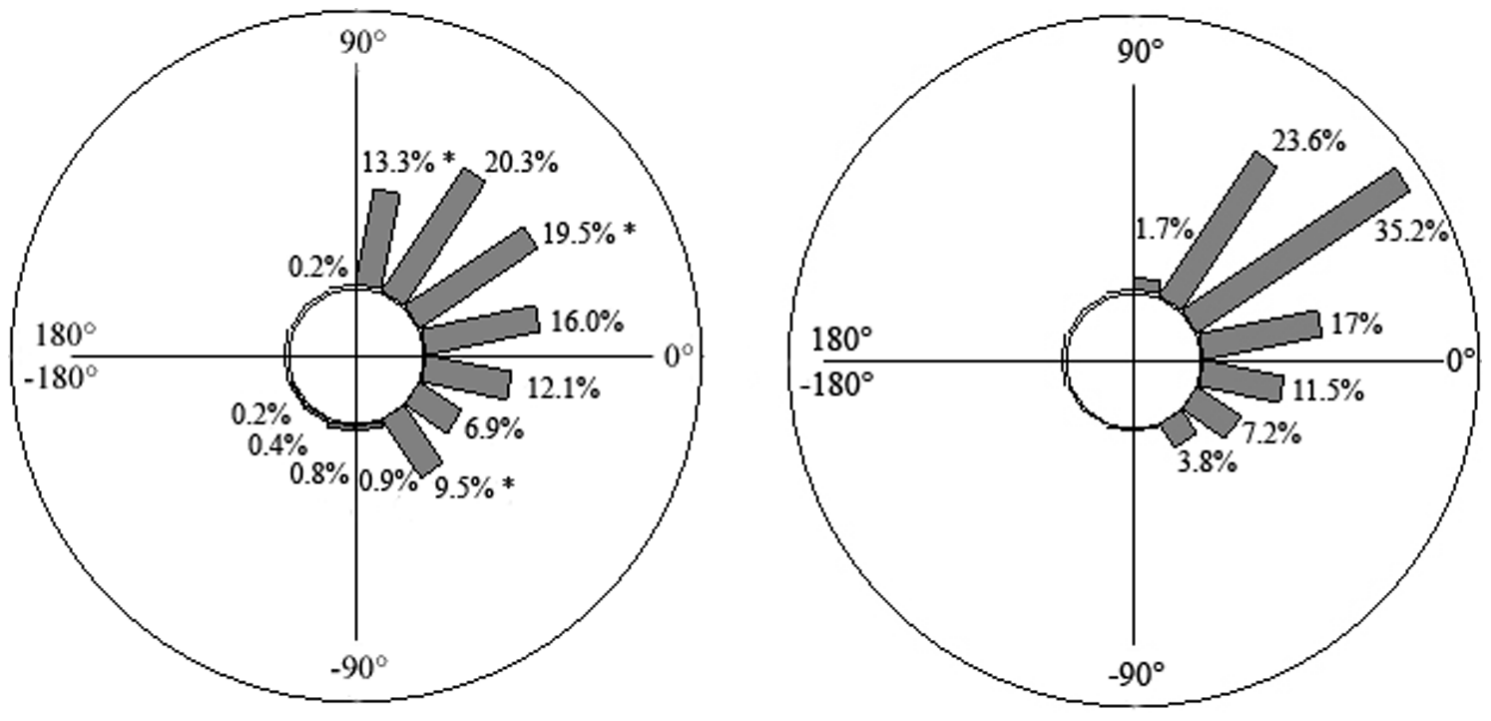
Lower limb coordination: Time spent (in % of the climbing duration) with the crampons in different angular positions; left panel: expert climbers, right panel: beginners; *: significant differences with beginners at *p*<0.05.

Typically during the whole climb, both groups tended to keep their two ice tools horizontally located (12.6±21.3° for beginners and 13.1±15.8° for experts) and their crampons in a horizontal position (22.2±16.2° for beginners and 18.8±20.7° for experts). However, experts tended to vary the angular positions of their ice tools more (standard deviation of ice tools angle = 48.6±6.6° *vs.* 27.1±7.6° for beginners; F_1,6_ = 27.25, *p*<0.05) as well as their crampons (standard deviation of crampons angle = 24.2±9.8° *vs.* 36.9±12.6° for beginners; F_1,6_ = 7.49, *p*<0.05), exploring a larger range of angular positions: from −135° to 180° for upper limb coordination and from −157.5° to 112.5° for lower limb coordination. Conversely, beginners tended to explore angular positions from −90° to 112.5° for upper-limb coordination and from −67.5° to 90° for lower-limb coordination (Fig. 3 and 4). Upper limb behaviours mainly differentiated climbing skill, since the expert group displayed differences with beginners for seven angular positions. In contrast, the two groups were different for only three angular positions in lower-limb coordination patterns (Fig. 3 and 4). Notably, beginners spent more time with their two ice tools and their two crampons diagonally located (respectively, 64.1±10.6% *vs.* 50.5±6.9% for experts, F_1,6_ = 8.62; 69.8±13.9% *vs.* 56.1±9.2% for experts, F_1,6_ = 10.32, all *p*<0.05) and less time with their ice tools and their crampons in a vertical position compared to experts (respectively, 2.4±3.9% *vs.* 21.3±9.4% for experts, F_1,6_ = 15.91; 1.7±3.1% *vs.* 15.2±12.7% for experts, F_1,6_ = 8.41, all *p*<0.05). Moreover, unlike beginners, the experts moved their ice tools (1.2±0.9%) and their crampons (0.6±1.5%) across the vertical mid-line of their bodies, explaining the larger variety of angular positions they attained during performance.

### Types and Frequency of Ice Tool and Crampon Actions

In experts the ratio between swinging or kicking actions and hooking actions was 0.6±0.2 for ice tools and 0.8±0.2 for crampons, which were significantly lower ratio values than displayed by beginners (ratio between ice tool swinging and hooking was 1.7±0.7, F_1,6_ = 8.35, *p*<0.05; and ratio between crampon kicking and hooking was 3.2±0.5, F_1,6_ = 17.23, *p*<0.05). Concerning the frequency of actions used to anchor the tools, experts revealed a ratio between swinging or kicking actions and definitive anchorages of 0.6±0.3 for ice tools and 0.9±0.2 for crampons, which were significantly lower values than observed in beginners (ratio between ice tools swinging and definitive anchorage was 3.6±0.4, F_1,6_ = 18.27, *p*<0.05; and ratio between crampons kicking and definitive anchorage was 4.7±0.6, F_1,6_ = 23.65, *p*<0.05).

### Perceptions, Actions, and Intentions Meaningful for the Climbers

In this section we examine some experience descriptions corresponding to perceptions, actions and intentions of beginners and experts. The most important environmental properties highlighted by both experts and beginners are the steps and holes in the ice fall (e.g., beginner 1: “*I put my ice tool in holes, in easy holds…it means there are a lot of crevasses in the ice surface easy to find*”; expert 1: “*the first thing I look is where I will anchorage my ice tools, so I’ll globally look for holes*”). However, our results indicated differences in hole perception between experts and beginners leading to different emergent actions (Tables 1 and 2). Through their perception and actions, both beginners and experts actualized intentions to focus on safety, but experts’ intentions mostly related to efficiency and energy economy (Tables 1 and 2).

## Discussion

In accordance with previous findings of Cordier et al. [38], who showed the acceleration of the centre of gravity as harmonic in experts and stochastic in beginners, during ascent of the ice fall our sample of expert climbers showed a more homogeneous trajectory of vertical pelvis displacement, while beginners showed an alternation between climbs and pause (i.e., plateaux). The longer plateau durations of the beginners could have been due to several reasons, particularly fear of falling, which could have led them: (i) to vary their coordination patterns less, whereas experts exhibited functional movement variability, a sign of inherent neurobiological system degeneracy, and (ii), to exploit environmental constraints less than experts who showed more perceptual attunement and calibration to functional informational variables (such as holes in icefall) specifying effective actions.

### Expertise Related to Functional Coordination Pattern Variability

**Figure 5:**
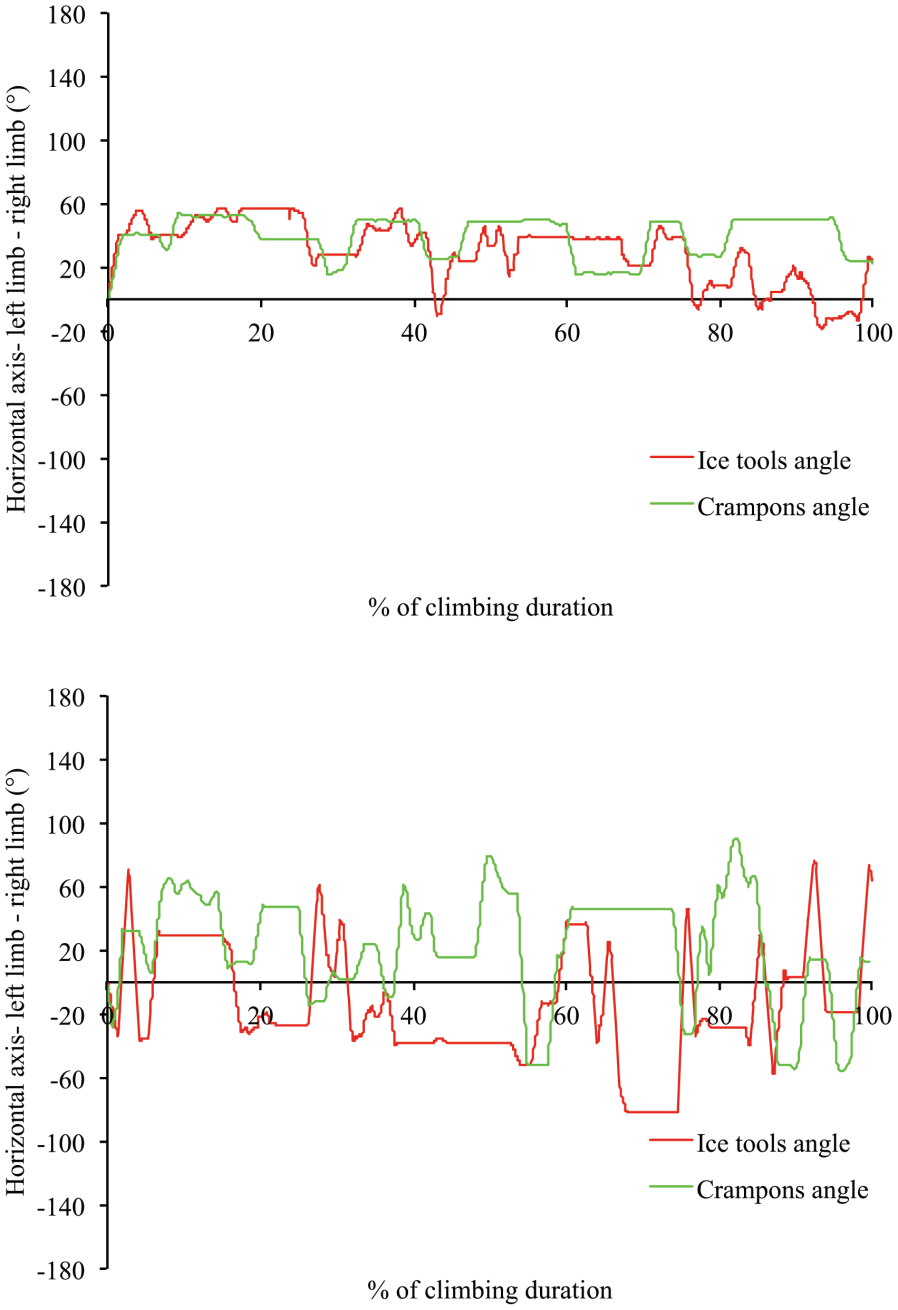
Example of angle-time curve for ice tools and crampons angle of two beginners showing numerous plateaus (5a: Participant 1 on top panel; 5b: Participant 2 on low panel).

**Figure 6:**
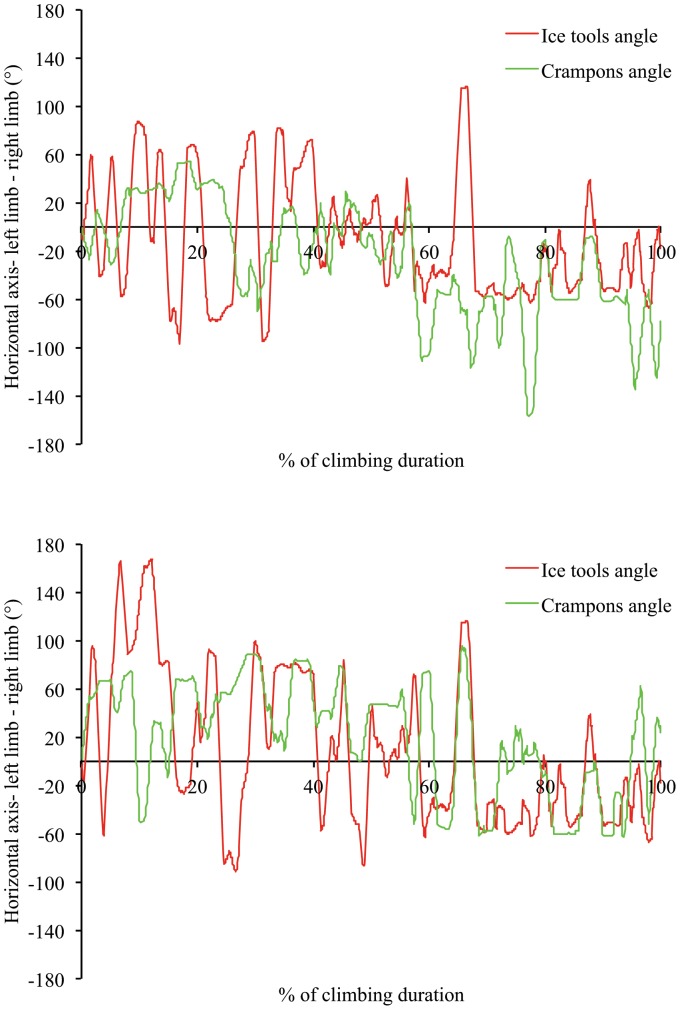
Example of angle-time curve for ice tools and crampons angle of two expert climbers showing high variability in the upper limb and lower limb coordination (6a: Participant 3 on top panel; 6b: Participant 4 on low panel).

Although no differences were observed between experts and beginners in upper-lower coordination interactions, beginners varied their upper limb- and lower limb-coordination patterns much less than the experts. Beginners mostly used horizontally- and diagonally-located angular positions (since limb anchorages are at the same level for the horizontal angle, the arms or legs appear in an in-phase coordination mode) (Figure 5 displays two examples of an angle-time curve of beginners). This strategy of motor system (re)organization led them to maintain a static “X” body position with arms and legs extended or with arms flexed and legs extended, which was likely to be achieved at a high energy cost and resultant fatigue, due to long periods of isometric muscle contraction [57]. Conversely, experts exhibited multi-stability in their inter-limb coordination patterns (e.g., horizontal-, diagonally-, vertical- and crossed located angular positions) to achieve their task goals (Figure 6 displays two examples of an angle-time curve of experts). According to the results of the self-confrontation interviews (Table 1), high levels of coordination pattern variability reflects functional adaptation to environmental properties; in particular the location of the anchorage and the type of movement used to anchor the ice tools and crampons were selected to save the icefall structure which looked fragile in some parts. Thus, the multi-stability of coordination patterns revealed an efficient environment-performer coupling in experts that was likely predicated on their inherent neurobiological system degeneracy [11], [12] and affordance perception. Indeed, the variety of coordination patterns is accompanied by various types of movement as experts are able: (i) to swing the ice tools and to kick the crampons in many different ways, often adapting the frequency of actions in relation to ice density and thickness; and (ii), to alternate swinging and hooking their ice tools and crampons into already existing holes (i.e., exploiting information on the shape of the ice fall).

### Expertise Related to Perceptual Attunement and Calibration of Informational Variables

It is important to recall that perceptual attunement and calibration are not synonymous concepts in ecological psychology. According to Fajen et al. [21]: “*Attunement refers to changes in the informational variables upon which one relies. A performer can be properly attuned, in the sense that he is relying on an informational variable that invariantly specifies the relevant property, but not properly calibrated. Thus, to reliably perceive affordances, both attunement and calibration are necessary*” (p.98). In our study, beginners seemed mostly attuned to visual characteristics of the icefall, as they focused on the size and depth of holes and steps, but observations suggested that they were not properly calibrated. Beginners exhibited a global perception of icefall shape for which big and deep holes in icefall were synonymous with deep and confident anchorages. This unique perceptual approach to the icefall led them to infrequently vary their limb coordination modes, and in particular to exhibit a tendency towards a static quadruped position that allowed them to maintain their equilibrium, with respect to gravity, under control (as observed in previous studies of rock climbing; Bourdin et al. [58]. The lack of calibration was revealed since beginners tended to infrequently vary the type of actions that they relied upon, repeating numerous ice tool swinging actions and crampon kicking actions in order to create deep holes and maintain confidence in their anchorages. Although this behaviour might enhance their stability on the icefall, it also led them to greater levels of fatigue and, as observed in a repetitive hammering task [59], to a highly specific type of motor system organization (e.g., a long time spent in a static “X” body position). The repetitive ice tool swinging behaviour was close to that observed in studies of rock climbing showing that, if the frequency of holds is equal to or greater than three, climbing ascent is slow, because equilibrium is always under control [27], [42] and climber tended to engage in numerous exploratory movements before displaying performatory movements [49]. Conversely, if climbers use a smaller frequency of holds to move up an artificial climbing wall, attunement and calibration are revealed by a functional mix of stability and instability, since they have to be quick enough to maintain equilibrium on the wall [27], [42].

Our results also emphasized the more heightened perceptual attunement of expert climbers to visual, acoustic and haptic information, which acted like specifying information sources that allowed climbers to detect use-ability of holes in the icefall. It must be recalled that detecting a certain information source that specifies a property of the environment allows the performer to make reliable judgments about this property [6]. In our study, the attunement to icefall thickness, density, shape and steepness that the climber experienced through his ascent may specify hole properties and how the climber might interact with this hole (e.g., ice tool swinging *vs.* hooking; one swinging *vs.* numerous swinging). Moreover, unlike the beginners, the more heightened perceptual calibration of expert climbers seemed to emerge from the embodiment of the ice tool [60]–[62]. The ice tool seemed like an arm extension [62] that allowed the climber to actively explore hole depths and orientations with the blade of the ice tool, and to experience the density and thickness of the ice around each hole, notably through the pick up of acoustic information from the blade during anchoring. Thus, the better calibration to environmental properties by the expert climbers highlighted their more effective perception-action coupling and their better perception of affordances which can invite a mix of functional behaviours [26]. According to Withagen et al. [26], “*affordances are not as invitations or solicitations to act, but as action possibilities that can invite*” (p.255). These authors recalled Gibson’s proposal, suggesting that invitations to act were viewed from a mutualist perspective, neither as subjective (agent properties) nor objective (environment properties) [20]. Rather they depended on the relation between the physical properties of the environment and each performer. In our study, holes in the icefall can invite behaviours, depending on hole properties (e.g., size, deepness, orientation) and climber properties (e.g., multi-stability of limb coordination patterns and types of action displayed). Climbing skill level and past experiences seemed to influence the nature of specific environment-performer couplings and the manner of perceiving affordances, since only experts could vary their motor behaviours to save energy, balance their body and maintain constant climbing fluency and the speed of ascent. Indeed, while beginners displayed a global perception of holes that typically led them to only swing their ice tool until a deep anchorage was achieved, experts exhibited functional multi-stability of their coordination patterns and types of action (e.g., ice tool and crampons swinging and hooking). Invitations to swing or to hook existing holes is functional by avoiding repetitive ice tool swinging or crampon kicking, and enabled experts to save energy on the icefall. In fact, to interact as they did with key environmental information constraints, the expert climbers alternated horizontal, diagonal, vertical and crossed angular limb positions on the icefall surface, exploiting the functionality of intra-individual coordination pattern variability. Indeed, to achieve that level of performance, expert climbers sometimes moved their right and left limbs across the vertical mid-line of their bodies to exploit information and hook existing holes in the icefall. Boschker et al. [27] previously showed how expert rock climbers recalled more information and focused on the functional properties of a climbing wall, neglecting to perceive its structural features. Conversely, the beginners in their study were not able to recall such functional properties of the wall for action and they tended to report almost exclusively the structural features of the holds [27]. The data from our study confirmed the importance of the functional properties of surface holes in ice climbing and suggested that holes in the icefall (like holds in rock climbing) could act as affordances that can invite behaviour of ice climbers during an ascent. In conclusion, the adaptive behaviours of expert exemplified how multi-stability of coordination patterns and types of movement is predicated on neurobiological degeneracy as well as the capacity to perceive affordances. Finally, our results highlighted that in exploiting degeneracy, expert climbers were also opening up further opportunities to detect affordances and vice versa, suggesting that affordance perception and neurobiological degeneracy may evolve together to support skilled performance.
